# Deep learning models to map osteocyte networks from confocal microscopy can successfully distinguish between young and aged bone

**DOI:** 10.1371/journal.pcbi.1013914

**Published:** 2026-01-27

**Authors:** Simon D. Vetter, Charles A. Schurman, Tamara Alliston, Gregory Slabaugh, Stefaan W. Verbruggen

**Affiliations:** 1 School of Electronic Engineering and Computer Science, Queen Mary University of London, London, United Kingdom; 2 Digital Environment Research Institute, Queen Mary University of London, London, United Kingdom; 3 Department of Orthopaedic Surgery, University of California, San Francisco, California, United States of America; 4 UC Berkeley/UCSF Graduate Program in Bioengineering, San Francisco, California, United States of America; 5 Buck Institute for Research on Aging, Novato, California, United States of America; 6 National Institute of Arthritis and Musculoskeletal and Skin Diseases, National Institutes of Health, Bethesda, Maryland, United States of America; 7 Alan Turing Institute, British Library, London, United Kingdom; 8 Centre for Bioengineering and Centre for Predictive in vitro Models, School of Engineering and Materials Science, Queen Mary University of London, London, United Kingdom; 9 INSIGNEO Institute for in silico Medicine, University of Sheffield, Sheffield, United Kingdom; King's College London, UNITED KINGDOM OF GREAT BRITAIN AND NORTHERN IRELAND

## Abstract

Osteocytes, the most abundant and mechanosensitive cells in bone tissue, play a pivotal role in bone homeostasis and mechano-responsiveness, orchestrating the delicate balance between bone formation and resorption under daily activity. Studying osteocyte connectivity and understanding their intricate arrangement within the lacunar canalicular network is essential for unravelling bone physiology, which is significantly disrupted during ageing. Much work has been carried out to investigate this relationship, often involving high resolution microscopy of discrete fragments of this network, alongside advanced computational modelling of individual cells. However, traditional methods of segmenting and measuring osteocyte connectomics are time-consuming and labour-intensive, often hindered by human subjectivity and limited throughput. In this study, we explored the application of deep learning and computer vision techniques to automate the segmentation and measurement of osteocyte connectomics, enabling more efficient and accurate analysis. For this specific application, once trained, the analysis was completed within 10 seconds, compared to manual segmentation time of 130 hours. We compared a number of state-of-the-art computer vision models (U-Nets and Vision Transformers) to successfully segment the osteocyte network, finding that an Attention U-Net model can accurately segment and measure 81.8% of osteocytes and 42.1% of dendritic processes, when compared to manual labelling. While further development is required, we demonstrated that this degree of accuracy is already sufficient to distinguish between bones of young (2-month-old) and aged (36-month-old) mice, as well as partially capturing the degeneration induced by genetic modification of osteocytes. Comparison of the model predictions with manual measurements showed no significant difference, indicating that, with additional training, such deep learning algorithms could be trained to human-level accuracy when measuring the osteocyte network. By harnessing the power of these advanced technologies, further developments will likely shed light on the complexities of osteocyte networks with ever-increasing efficiency.

## Author summary

Osteocytes make up the vast majority of our bone cells, and are spread in a complex network throughout our bone tissue, making them difficult to visualize and time-consuming to measure. This study applied cutting edge machine learning techniques to confocal microscopy scans of the osteocyte network, cutting analysis time down from 130 hours to 10 seconds. Interestingly, the models were sufficiently accurate to distinguish between bones of young and aged mice. Similarly, the models partially captured the degeneration induced by key genetic mutations. This study presents a powerful new tool to analyse bone structure at the cellular scale.

## Introduction

Osteocytes are the mechanosensitive cells underlying the exquisite ability of bone to finely balance formation and resorption in response to daily activities [1]. These cells are spread throughout bone tissue, residing within a vast system of lacunae and canaliculi. This system, known as the lacunar-canalicular network (LCN), forms a network of intricately connected architecture that is crucial for maintaining nutrient supply for osteocytes distant from the vasculature [2]. Within the LCN the osteocyte network processes biochemical signals that regulate bone matrix production [3], mineralisation [4] and resorption [5] such that skeletal strength is maintained. This network is now considered to act as a vast endocrine organ, as it is proposed to regulate processes as varied as myelopoiesis [6], as well as glucose metabolism [7], inflammatory signalling [8,9] and fertility [10] via secretion of osteocalcin and other cytokines.

By far the most abundant bone cell type, osteocytes act as a network of strain gauges, with the connections between them allowing the integration of signals to the osteoblasts and osteoclasts at the bone surface [11]. Furthermore, as osteocytes are highly mechanosensitive, the local architecture of the osteocyte network (dendrite number, connectivity, osteocyte density, canalicular spacing, perilacunar-canalicular remodelling) immediately surrounding individual osteocytes can affect their mechanosensitive response [12–15] and, with growing evidence that osteocytes have the ability to alter this local geometry themselves [16–18], it is also possible that they may tune their local environment to maintain homeostatic stimulation [19]. Perhaps most indicative of the importance of this osteocyte network, recent studies have found significant disruption to this system occurs alongside broader degeneration of properties in aged bone, with degradation of the osteocyte network [20], including loss of canaliculi and changes in lacunar geometries [21–23]. Similarly widespread changes both the morphology of the osteocyte network and osteocyte mechanosensitivity occur in diseases such as osteoporosis [24–26] and metastatic lesions [27]. While connectomics techniques do exist in other biological fields (e.g., neurobiology), in recent years they have begun to be applied to the osteocyte network, which allows some degree of automation by deploying a range of processing steps. A broad un-biased connectomics analysis of the lacunar-canalicular network in mice and sheep was conducted without manual segmentation, finding that osteocyte network properties appear to be conserved across bone types and mammalian species [28]. Spatial network analysis using limited manual intervention has also been deployed to assess lacunar-canalicular network integrity [29,30], and how this changes in ageing in mice [19,31]. In particular, based on the connectomics code in our previous study [19], Yilmaz et al. have proposed an effective contrast enhancement approach that dramatically improves thresholding quality [31], with the open-source code freely available. Since robust quantitative analysis of the osteocyte network is critical to understanding the role and regulation of osteocytes in bone health, aging, and disease, the continued reliance on either time-consuming manual segmentation or multiple pre-processing steps remain limiting factors for the field. Therefore, what is clearly still required is a rapid and automated analysis tool, which can overcome incorrect labelling of imaging artefacts without manual adjustment of parameters for each image, to segment and measure osteocyte connectomics, in disease and health.

**Fig 1:**
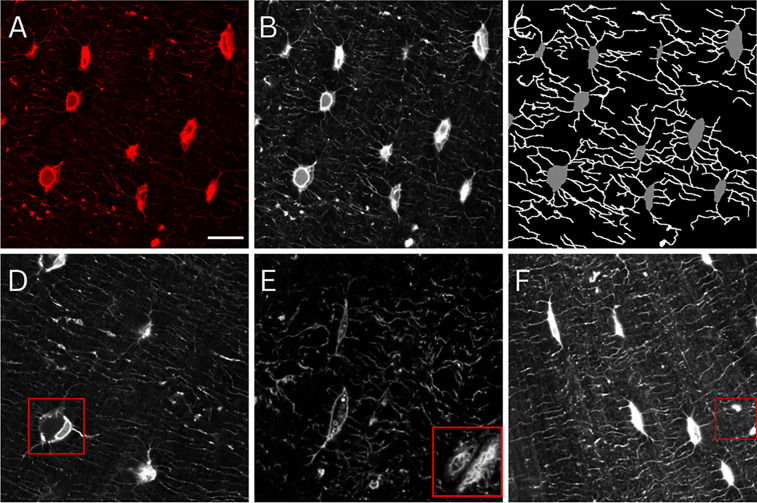
Osteocytes and dendrites manually labelled to train deep learning models. (A) Confocal laser scanning microscopy images of osteocytes (red, phalloidin, cytoskeleton). (B) Images were greyscaled for training, (C) followed by manual annotation and labelling of osteocytes and dendrites. Raw images contained features that presented significant challenges to deep learning models, including (D) shrunken osteocytes possibly fixed during apoptosis,(E) transiting blood vessels, and (F) locations of fused dendrites or the edge of an out-of-plane osteocyte. Scale bar = 10 µm and applies to all panels.

The significant recent advances in machine learning models in biomedical imaging present a promising opportunity for osteocyte researchers. Deep learning models, such as Convolutional Neural Networks (CNNs), are now the foundation of numerous high-performing medical image segmentation models [32]. In particular, U-Net, developed by Ronneberger et al. [33], has gained widespread acceptance in biomedical image segmentation due to its ability to generalise across diverse tasks and perform effectively even with limited amounts of labelled data. However, due to the limited kernel size and inherent locality of the convolution operation, these CNN-based approaches have exhibited limitations in learning long-range dependencies and global context, which are vital for segmenting complex datasets [34]. The recent successes of Transformers in the Natural Language Processing (NLP) [35] field has inspired researchers to adapt this novel method to the vision domain. These models employ self-attention mechanisms to construct comprehensive encoder-decoder architectures, facilitating the capture of long-range dependencies inherent in image data. Numerous studies have integrated the transformer into medical image segmentation [32], exhibiting results that either match or exceed the current state-of-the-art [32]. Additionally, the Swin-UNet model leverages the adaptability and self-attention benefits of transformers along with the localised feature extraction capabilities intrinsic to the U-Net design [34]. This hybrid approach showcases the potential of integrating the strengths of different architectural designs to improve the performance of image segmentation tasks. Nevertheless, image segmentation remains a formidable challenge. While a number of models have been trained in monolayer cultures for *Kerschnitzki (2013)* analysis of cells and nuclei [36–39], the intricate 3D nature of the *in vivo* lacunar-canalicular network likely requires bespoke training. Particularly, in the context of the osteocyte network, semantic segmentation encounters several unique obstacles. These include image noise from microscope images, varying illumination and staining conditions in cellular microscopy (due to irregularities of dye absorption), irregularities in cell shapes, and the presence of abnormalities within the data (Fig 1).

Perhaps due to these challenges, to date, a truly machine learning approach has not been successfully applied to the segmentation of the osteocyte network. Thus far, as will be discussed in the following paragraphs, researchers have relied on thresholding or region-growing to segment the osteocyte network, which often require manual intervention to correct artefacts and remove background, sometimes pixel-by-pixel. Several approaches can also only handle one set of 2D images at a time, or one region at a time, and are not suitable to analyse a large portion of the osteocyte network (i.e., images containing more than 3 osteocytes). Kerschnitzki et al. employed a low-pass filter and adaptive thresholding to segregate the LCN [30], though this approach is susceptible to image artefacts and capturing objects of larger size (e.g., blood vessels). Building on this to analyse connectomics of the LCN, Mabilleau et al. employed a thinning algorithm to refine the dendrites into a singular-voxel-wide skeleton [40], and then computed the length and degree distributions of the network. Further application of this technique required meticulous manual examination to detect and manually draw in overlooked osteocytes and dendrites [41]. Similar semi-manual approaches were applied by other researchers, attempting to detect and segment osteocytes by fitting ellipsoids to cell bodies [42,43]. Additional work by Heveran et al. also incorporated ellipsoid shape priors and thresholding to develop an automatic segmentation and analysis technique of osteocyte lacunae within 3D images from confocal laser scanning microscopy (CLSM) [23], a popular imaging method for obtaining images of the canalicular network via optical sectioning at sufficient resolution to allow 3D reconstruction [44,45]. The model demonstrated notable predictive efficacy, achieving accurate segmentation of 77.1–97.8% of the lacunae in their experimental dataset [23]. Most recently, Weinkamer and colleagues have developed an elegant technique to segment the lacunar-canalicular network in 3D [29], along with quantitative connectomic analysis of osteocyte lacunar-canalicular networks in three dimensions. Nevertheless, akin to other thresholding methodologies, these image pre-processing procedures require manual adjustment of filtering and thresholding parameters when applied to different datasets.

A more complex automated segmentation method, variational region growing (VRG) [46], has also been applied to the LCN, however it was initially limited by segmenting osteocytes and dendrites into the same class [47]. Therefore, despite some early attempts, computer vision and machine learning methodologies have yet to be fully applied to efficiently measure the intricate architecture of the osteocyte network.

Given the biological complexity and computational challenges described, we first compared a range of automated thresholding methods with those applied previously, with the aim of identifying the most successful. Next, we trained a number of CNN models on confocal microscopy images of osteocytes, improving and challenging their accuracy until a successful model was identified. In order to demonstrate the scientific and clinical potential of these techniques, we tested the hypothesis that the model can distinguish between young and aged bone. Finally, this model was challenged to distinguish bone from genetically modified mice known to have osteocyte network degeneration from their wild-type controls.

## Materials and methods

### Ethics statement

All animal procedures were approved by the Institutional Animal Care and Use Committee of the University of California San Francisco (UCSF) and the Buck Institute for Research on Aging [19].

### Fluorescent imaging of mouse osteocyte networks

Confocal laser scanning microscopy (CLSM) was applied to mouse bones from our previous ageing study. Here, we collect stacks of 2D confocal images to provide data to train our 2D image analysis algorithm. Femurs for cryosectioning and fluorescent imaging were prepared as previously described [19,22,48,49]. Briefly, bones were dissected to remove surrounding tissue, including periosteum, followed by fixation in 4% PFA for 24–48 hours [49]. Bones were then rinsed in PBS, and decalcified in 0.5M EDTA for 2–4 weeks on shaker at 60 revolutions/ minute at RT. EDTA solution was changed every 3–4 days. Following decalcification, bones were rinsed in PBS before cutting of ends and gently removing bone marrow by flushing with PBS using a syringe and needle. Samples were again rinsed in PBS and then subjected to a sucrose gradient (10%, 15%, 20% for 15 minutes at RT) followed by a 30% sucrose solution overnight at 4°C [49]. Samples were then dried and frozen embedded in OCT media as per standard protocol. Cryosections 40–100 μm thick of the embedded bones were cut, either onto microscope slides or wells of a 48 well plate containing ~250 μl RT 1X PBS for further rinsing before staining and mounting as per standard protocols. Staining of the osteocyte network was accomplished utilising the hydrophobic lipophilic dye, DiI (ThermoFisher), Alexa Fluor 488-Phalloidin to visualize the actin cytoskeleton (ThermoFisher), and DAPI for identification of cell nuclei. Bone sections were optically cleared and imaged on a Leica DMi8 (Leica Microsystems) inverted microscope running LASX software, and a comprehensive protocol for this method was described by Dole et al. [49].

Single femur bones from inbred, male C57BL/6 mice (young, 2 months, N = 4 and aged, 36 months, N = 5) from the Buck Institute for Research on Aging (Novato, CA) were used for comparisons regarding age-related phenotypes, with genetic modification-induced degeneration measured in femurs from male (young, 2 months) TβRII^ocy-/-^ mice and their TβRII^ctrl^ littermates (N = 4–5 ea.) [19].

Regions of interest (ROIs) of 75 μm x 75 μm x ≥ 35 μm were captured for 3 independent regions around the cortex within lamellar bone for each sample at a 500x500 pixel resolution in the XY plane, resulting in a digital pixel resolution of 150 nm/pixel. Given the optical wavelength of the fluorophores used, we were practically diffraction limited to a resolution of ~450 nm, constraining analysis to features greater than ~3 pixels in width.

While z-stacks of multiple osteocyte networks in 3D were available, these were not applied in this study due to computational cost and high error rates in initial trials. Instead, individual images of the osteocyte network were collected across multiple randomly selected regions in the femur, resulting in approximately 100–135 images per group for testing the model.

### Datasets

In order to successfully segment the osteocyte network, a process of labelling, manually annotating features within images to identify them, i.e., as an osteocyte cell body, or an osteocyte dendritic process, was required. Although the initial collection of multiple z-stacks consisted of 1,224 individual 2D images, taken from the bones of healthy wildtype mice, constraints in labelling (e.g., images with only a single osteocyte or images that were too dark to label accurately) reduced the number of images suitable for training (892 images). In this study, we utilised two distinct datasets for initial model training and selection, followed by further training of the most successful model on an enlarged dataset. This required manual annotation and review of every image by two operators, one expert (>15 years expertise) and one trained, taking a total of 130 hours of manual labelling. A final dataset of young, aged, and genetically modified mice was then analysed for comparison with a human operator.

#### Dataset 1.

This comprised a manually annotated set with 30 training and 7 validation images (each one a 2D image from a different available z-stack). The manual labelling was facilitated by the automated image analysis tool, arivis Cloud (Carl Zeiss Microscopy GmbH, 2023).

#### Dataset 2.

This dataset comprised 696 training images alongside 196 images designated for validation (i.e., a training dataset and a test dataset). In order to build a large training dataset. Dataset 2 comprised individual 2D images from different locations in the same z-stack, for multiple z-stacks of multiple mice. In each case individual images were separated by more than 100 μm to ensure that no osteocyte was used for training more than once. The labels within this dataset were derived from a segmentation model previously trained on Dataset 1. As a result, these labels inherently possessed certain biases and were primarily employed for training with partial labels, which allowed the model to select a true label from either Osteocyte or Dendrites. The segmentation model’s automatic training was facilitated using the arivis Cloud software.

#### Dataset 3.

An additional separate 49 images (one 2D image from each individual available z-stack) were annotated manually in order to further train the Attention U-Net.

#### Dataset 4.

In order to compare the effectiveness of connectomics analyses by the model with a human operator, the model was applied to additional data. These comprised 135 images of young mice bone and 135 of aged mice, as well as 85 images of young TβRIIocy-/- and 102 images of TβRIIctrl mice, (in each case a 2D image an individual available z-stack).

### Evaluation metrics

In order to quantitatively evaluate the performance of the segmentation methods, Dice score coefficient (DSC) and Jaccard index or Intersect over Union (IoU) were employed. Both DSC and IoU are among the most commonly used metrics in semantic segmentation [50]. The equations are shown in (1) and (2) respectively where *P* - represents Prediction and *G* - Ground Truth. Dice metric evaluates the spatial overlap accuracy between the predicted segmentation and the ground truth. The Jaccard index (or IoU) is defined as the area of the intersection between the prediction and ground truth, divided by the area of the union of two label sets [50].


$$\text{DSC}=\frac{\text{2}|P\cap G|}{\left|P|+|G\right|}$$ (1)

$$\text{IoU}=\frac{\left|P\cap G\right|}{\left|P\cup G\right|}$$ (2)

### Segmentation using thresholding

We replicated the segmentation methods based on CLSM images described in Kerschnitzki (2013) [30], which we have termed M1; and those of both Mabilleau et al. [40] and in Ashique et al. [41], which we have termed M2. Owing to considerable variability in our data, such as bone sample preparation, spatial resolutions, dyeing agents etc. we optimised method parameters and threshold functions to our dataset. In addition to this, inspired by previous literature, we derived two additional threshold-based segmentation techniques. While both methodologies shared the same pre- and post-processing stages, they differed in their core segmentation techniques: one employed Canny edge detection for canaliculi segmentation, while the other opted for Otsu thresholding. The pre-processing began with a SciPy’s Gaussian filter with a standard deviation of 2 to decrease noise such that derivative-based thresholding functions were better facilitated. Secondly, we manually fit a binary threshold of 70 (on an image intensity scale of 0–255) to separate the image into two masks, the lower end being osteocytes and the higher threshold being dendrites. We performed a second binary threshold on the dendrite mask to remove additional noise. Core segmentation techniques were then applied (Canny(p1 = 70, p2 = 220) and Otsu(p1 = 80, p1 = 255)). The post-processing employed a morphological closing operation on the dendrite mask using a 3x3 kernel. This facilitated the refinement of segmented dendrites. Lastly, overlapping osteocyte and dendrite labels were removed using an elementwise subtraction yielding the final segmentation.

### Segmentation using deep learning models

In the realm of medical image segmentation, certain architectures have risen to prominence due to their superior performance on a myriad of tasks. In particular, U-Nets and Vision Transformers have recently been recognised for their state-of-the-art segmentation capabilities [51–53], including quantifying bone features via X-ray and CT, in pediatric and elderly patients [51–53]. As a brief explanation of these operations, the main principle underpinning the U-Net is the encoder and decoder components of the algorithm. The encoder works as the feature extractor and compresses the input image into a lower-dimensional representation, with the computer learning an abstract representation of the input image. Following this, the decoder reconstructs the compressed representation back to the original image size with the specified number of predicted features. The decoder involves layers that successively increase the size of the image while decreasing the number of features, essentially reversing the process of the encoder. While a standard U-Net relies on convolutional layers, Swin-UNet uses a type of layer called a transformer layer that incorporates self-attention. This mechanism allows the model to focus on different parts of the image more flexibly, learning which parts of the image are most important for the task at hand. As an illustrative example, if the model is trying to predict (i.e., locate) an osteocyte in an image, self-attention helps it focus on areas of the image that provide useful context for making that identification, such as multiple dendrites converging on one point. Guided by this knowledge and the prevailing literature, our experiments primarily centred on leveraging these architectures for our specific segmentation requirements.

#### Image preprocessing and augmentation.

Given that our RGB images only held information in the red channel, they were converted to grayscale to retain essential data while simplifying the input. To bolster data diversity and counteract potential overfitting of the models, various data augmentations, such as random flips (i.e., flipping a figure through its vertical or horizontal axis), rotations, and cropping, were applied. Additionally, gradient norm clipping was incorporated, and datasets underwent normalisation before training.

#### Training parameters and strategy.

Initial hyperparameters were retrieved from the original publications associated with each model [33,54–56]. However, to ensure optimal performance on our specific datasets, these parameters underwent meticulous testing and subsequent adjustments. For our best performing models, we applied PyTorch’s implementation of the AdamW optimiser with learning rate = 0.001, weight decay = 1e – 4 and cosine annealing scheduler with parameters T max = 10% of total steps and eta_min = 5e − 6. For the loss function, we used a weighted sum of Dice loss and Cross Entropy loss: DiceCELoss. The mentioned preprocessing techniques and training parameters were used for all experimental training in this project. For the best performing AttentionUNet model: Model configurations using Monai (Medical Open Network for AI) [57] AttentionUnet: spatial_dims = 2, in_channels = 1, out_channels = 3, channels = (16, 32, 64, 128, 256), strides = (2, 2, 2, 2). Specific data augmentations included random vertical and horizontal flip with p = 0.5, random rotations 30°, and random cropping with size 448. Training was conducted using the Andrena HPC facility at Queen Mary University of London. The computational tasks were executed on a single Nvidia A100 GPU.

#### Comparison of Dataset 1 and Dataset 2.

The first experiment adopted the following architectures: U-Net, UNet++, RegU-Net, Flex U-Net, SwinUNet, and UNETR [33,54–56]. These models were sourced from Medical Open Network for AI (MONAI), a PyTorch-based library tailored for medical imaging applications, with these having been deployed for similar segmentation tasks elsewhere [58]. These models were trained using two different training strategies: Strategy 1 where models were exclusively trained on Dataset 1, and Strategy 2 where initial training was conducted on Dataset 2, followed by fine-tuning on Dataset 1. The second strategy was implemented due to the time-consuming and challenging task of annotating 2D images from the original 3D stack of CLSM images (Fig 1). Notably, during the training on Dataset 1 in Strategy 2, the labels were partially annotated, hence, the loss function was exclusively computed over the annotated areas. As a result of this, we could leverage the segmentations predicted by a model trained on arivis Cloud on Dataset 1, thus giving access to a greater number of labels. The motivation behind this was to explore if increasing the dataset with sub-optimal labels could provide a better result compared with a much smaller training dataset but with better labels. The fine-tuning process in Strategy 2 was evaluated through three distinct strategies (commonly used in transfer learning for medical image analysis to mitigate overfitting on small datasets, leverage valuable pre-trained features, reduce computational costs, and prevent the destruction of general knowledge) [59]:

- Freezing all layers except input and classification layer
- Freezing all layers except classification layer
- Implementing a reduced learning rate on all layers, except the classification layer

#### Separating model prediction.

The model aimed to utilise mutual information and contextual cues from both osteocytes and dendrites in the image to learn intricate features. For instance, the gathering of numerous dendrites within a specific region could signify the presence of an osteocyte. Capturing such nuanced and interrelated features might necessitate a more extensive annotated dataset than was available to us. In light of these considerations, we also embarked on an alternative strategy: training two distinct models—one focusing on dendrite identification, and the other on osteocyte prediction. The aggregated outputs from these models yielded the final segmentation.

#### Encouraging longer and fewer dendrites.

Moreover, in order to leverage the models’ segmentation outputs for subsequent connectomics analysis, it was also imperative that the model was able to predict dendrites that establish clear interconnections between osteocytes. In order to encourage the model to output longer and interconnected dendrites, we incorporate an additional means of regularisation on the loss function as seen in S1 Appendix. This was done by first performing a connected components analysis of the dendrite prediction. We then calculated the size of each component and added an inverse penalty; smaller components led to larger penalties. The motivation behind this strategy was essentially to penalise the network for outputting short, isolated dendrites. Two specific models were employed for this endeavour: the CNN-based Attention U-Net and the transformer network Swin UNetR.

### Connectomics analysis

To facilitate a quantitative connectomics analysis of the segmentation outputs, the outputs were transformed into a graph representation using the Python library NetworkX. In this structured representation, osteocytes served as nodes, with dendrites forming the interlinking edges between them. To achieve this, first via manually testing and verification on simplified dummy data, the segmentations were initially separated into two binary masks: Osteocytes and Dendrites. Subsequently, a morphological dilation was applied to the masks using a 4x4 ellipsoidal structure for Osteocytes and a 2x2 cross-structuring element for Dendrites. This dilation procedure aimed to merge nearby components like noisy dendrite predictions that, ideally, should represent a singular entity. After dilation, a connected component analysis was executed on both classes to individually discern unique components. To establish edges that link osteocyte nodes, an iterative process was undertaken on dendrite segments, verifying overlap with osteocyte labels based on their x and y coordinates. This framework further facilitated the computation of metrics, encompassing the network’s degree, the shortest path between osteocytes, the frequency of terminus connections per osteocyte (dead-ends), and dendrite thickness across the network and individual connections. Connections were counted when there is an independent route available connecting two osteocytes cells that does not pass through another osteocyte alone, meaning that connections between two osteocytes via intermediate osteocytes were not counted.

### Comparison between measurements of manual and deep learning model

A comparison of connectomics analyses with manual operation was conducted by comparing the characterization of multiple conditions present in Dataset 4, with previous manual analyses of the same dataset [19]. Comparing the manual segmentation and quantification of the osteocyte network with the deep learning method was challenging, as the manual methods attempt to quantify osteocyte network parameters in 3D, while the deep learning model has thus far only been trained on 2D images. For example, the manual methods quantified ~100 canaliculi per osteocyte in 3D, while the deep learning models calculated ~30 in visible in each 2D image. Therefore, to investigate the capability of the model trained only on 2D images in approximating laborious 3D quantification, we compared the percentage changes quantified in 2D by the model, vs the percentage changed quantified manually on the 3D dataset. As the percentage decrease in each analysis must be calculated as a change in the average value for the one group compared to the average value for another group, statistical analysis could not be performed on these single values and they are instead presented in absolute terms.

### Statistical analysis

In order to test the success of the model at distinguishing between different experimental groups, statistical analyses performed using a non-parametric Mann-Whitney U test with a defined alpha level of 0.05 (statistical significance is indicated by * for *p* < 0.05, **** for *p* < 0.0001, n.s. for no statistical significance, on figures). Prism 8.4 (GraphPad Software Inc.) was used for all statistical comparisons. Unless otherwise stated, data is presented as mean ± SD, and groups are detailed in the figure legends.

## Results

### Otsu and Canny thresholding approaches give higher accuracy when segmenting the osteocyte network

Average automatic segmentation time, dice score, mean IoU (mIoU) and IoU per class is presented in Table 1 for each of the thresholding methods applied by the algorithm. M1 is based on previous thresholding methods of Kerschnitzki et al. [30], while M2 replicated the manual adjustments to thresholding segmentation applied by Mabilleau et al. [40] and in Ashique et al. [41].

**Table 1:** Segmentation time, Dice score, mean IoU and IoU for individual classes. The methods M1 and M2 are derived from (Kerschnitzki, 2013; Mabilleau et al., 2016) [30,40], and (Ashique et al., 2017) [41] respectively, Otsu and Canny represent our non-DL segmentation methods.

Method	Time	DSC	mIoU	Osteocyte	Dendrite
M1	**0.000s**	0.55	0.46	0.383	0.093
M2	2.200s	0.156	0.094	0.194	0.088
Otsu	0.028s	**0.637**	**0.517**	0.47	0.222
Canny	0.017s	0.622	0.493	**0.472**	**0.226**

When comparing these previously applied methods with Otsu and Canny approaches we developed, we found that Otsu and Canny both give higher accuracy values. While both of our methods thresholded osteocytes and dendrites with similar accuracies (~47% and ~22%, or 0.47 and 0.22 mIoU, respectively), the Otsu method gave the highest confidence, with a DICE score of 0.637 and thus was the most promising segmentation methods among the four thresholding approaches. While M1 had an advantage in execution speed, its segmentation performance lagged behind Otsu and Canny. M2 performed significantly worse in all metrics compared to the other methods.

### Attention U-Net is the most effective deep learning model capable of segmenting osteocytes and their dendritic processes

#### Comparison of training strategy 1 and 2.

Performance metrics of the models from the two different training strategies can be viewed in Table 2. Among the evaluated models, the Attention U-Net achieved the highest Dice score and mIoU for both strategies. In contrast, the RegU-Net model displayed notably lower performance than the other models in both the Dice score and mIoU. The performance of UNet and U-Net++ were quite similar for training strategy 1, and inconsistencies arose in strategy 2. The Vision Transformers (UNETR and Swin UNETR) performed less well, with lower DSC and IoU values than the other models. In particular, Swin UNETR’s performance was notably inconsistent between the training strategies. It performed well on Dataset 1 (only slightly below U-Net), but its performance dropped significantly in strategy 2.

**Table 2:** Performance metrics of implemented models on two different training strategies.

Models	Training strategy 1 (Dataset 1)				Training strategy 2 (Dataset 2 + fine-tune on Dataset 1)			
	DSC	mIoU	Osteocyte	Dendrite	DSC	mIoU	Osteocyte	Dendrite
U-Net	0.795	0.694	0.773	0.389	0.195	0.117	0.015	0.088
U-Net++	0.794	0.694	0.779	0.385	0.794	0.694	0.78	0.383
Attention U-Net	**0.801**	**0.703**	**0.793**	**0.394**	**0.8**	**0.702**	**0.794**	**0.391**
RegU-Net	0.523	0.432	0.393	0.05	0.504	0.431	0.402	0.005
UNETR	0.76	0.659	0.755	0.301	0.759	0.658	0.755	0.305
Swin UNETR	0.79	0.69	0.782	0.37	0.234	0.36	0.113	0.179

#### Experiments to improve dendrite segmentation accuracy.

**Fig 2:**
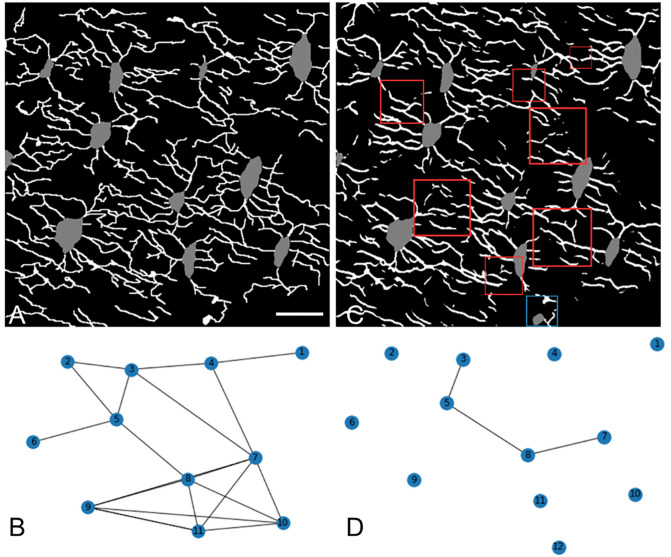
Connectomics used as training strategy for deep learning models. (A) Annotated labels in an image, and (B) the corresponding connectomics map. After training, model is tested to produce a (C) prediction and (D) corresponding connectomics map, which can be compared to the annotated map to assess accuracy. Red squares in the prediction highlight instances of disconnected dendrite segments, while the blue square indicates a misclassification where a dendrite has been erroneously labeled as an osteocyte. Scale bar = 10 µm and applies to all panels.

The outcomes of the three experiments—namely, separating model predictions, implementing a novel regularisation technique to promote larger yet fewer dendrites, and employing label dilation to account for annotation inaccuracies—are summarised in Table 3. The findings demonstrated that the initial two experiments exerted minimal impact on the segmentation accuracy of the dendrite class. In contrast, the approach involving the dilation of dendrite labels prior to training yielded a notable enhancement in dendrite accuracy, registering an increase of 25% for the Swin UNet and 22% for the Attention U-Net. Among the models examined, the Attention UNet consistently exhibited superior performance. Superficially, dendrite predictions appeared satisfactory when studying the segmentation results depicted in Fig 2. However, upon closer inspection, we observed the presence of fragmented and disconnected dendrite structures.

**Table 3:** Performance metrics of experiments attempting to improve dendrite segmentation accuracy (L-Reg: Local affine registration method).

Models	Dataset 1			
	Dice	IoU	Osteocyte	Dendrite
Separate Swin UNETR	0.794	0.693	0.779	0.381
Separate Att. U-Net	0.797	0.695	0.766	0.402
L-Reg Swin UNETR	0.793	0.695	0.802	0.363
L-Reg Att. U-Net	0.802	0.703	0.791	0.396
Dilation Swin UNETR	0.813	0.709	0.790	0.463
Dilation Att. U-Net	**0.823**	**0.721**	**0.792**	**0.480**

#### Connectomics analysis.

The connectomics analysis in Table 4 was conducted using segmentation predictions from the top-performing model highlighted in Table 3. This model exhibited a tendency to overestimate the number of nodes, predicting an average of 8.30 nodes as opposed to the 7.00 nodes present in the labelled data. Notably, the model’s predictions presented fewer dead ends per node, averaging at 14.9, in contrast to the labelled data’s 20.9. Furthermore, it significantly undervalued the average number of connections per node, registering a mere 0.41 compared to the 3.84 seen in the labels. From the analysis, we also saw that the model produced shorter and thicker connections than the labels. These findings were also reflected in the comparison of a label’s graph and a segmentation’s graph in Fig 2.

**Table 4:** Connectivity metrics computed from the connectomics analysis. The average is taken from the validation set consisting of 7 images.

Metrics	Prediction	Label
Average nodes	8.3	7
Average dead ends per node	14.9	20.9
Average connections per node	0.41	3.84
Average length of each connection	14.3 µm	55.6 µm
Average diameter of connections	0.53 µm	0.38 µm
Average diameter of network	0.48 µm	0.37 µm

Having now selected the Attention U-Net as the most successful model, an additional 49 images were manually annotated to increase the training dataset (Dataset 3). Demonstrating the labour-intensive nature of this manual segmentation, pixel-by-pixel labelling of these datasets by an expert operator took more than 130 hours. Upon re-training the model, the accuracy of osteocyte detection increased to 81.8%, while the accuracy of dendrite detection increased to 41.2% when compared to an expert operator (DSC, accuracy by quantifying the overlap between total number of dendrites labelled and operator ground truth). While the accuracy of osteocyte detection was very high, it remained unclear whether the lower dendrite accuracy would be sufficiently high to distinguish between two populations, e.g., young and aged bone.

### Deep learning models can successfully distinguish between young and aged, or healthy and degenerated, osteocyte networks without manual intervention

Work by the authors has previously demonstrated that aging disrupts the integrity and predicted function of the osteocyte network, using labour-intensive computational modelling techniques to manually segment and monitor these changes, and relating them to age-related changes in gene expression [19]. Given that we have identified several parameters of the osteocyte network, described below, that are quantifiably different in aging when compared to younger mice, we challenged our deep learning model to similarly distinguish between the two.

**Fig 3:**
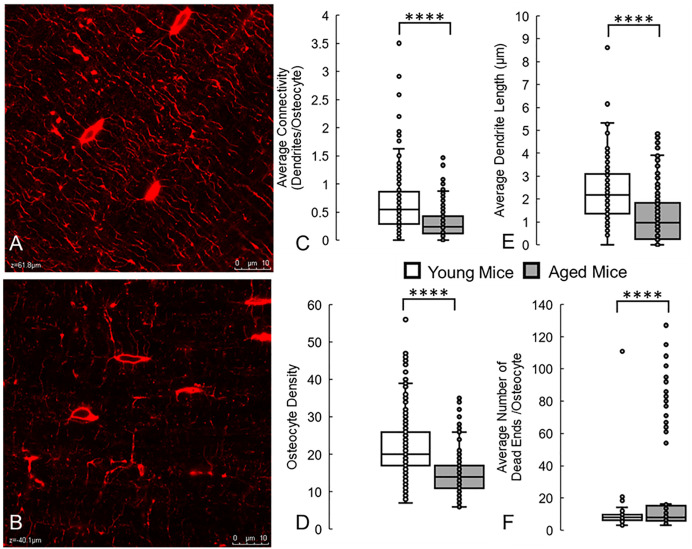
Deep learning model can accurately detect and identify the lower osteocyte connectivity of aged bone, when compared to young bone. These results are similar to changes we measured manually in a previous study [19]. (A) Confocal laser scanning microscopy images of osteocytes (red, phalloidin, cytoskeleton) from young (2 month old) and (B) aged (36 month old) mice. Running the trained deep learning model on these datasets correctly measured that aging causes significant decreases in (C) average network connectivity, (D) osteocyte density, and (E) average dendrite length. (F) The model also correctly predicted significant increases in blunted dendrites or dead ends in the network. **** denotes *p* < 0.0001 by Mann-Whitney U test compared to young control. Scale bar = 10 µm.

We found that the model was capable of correctly identifying and measuring the reduced connectivity of the network in 2D confocal microscopy scans of older mice, and the decreased average length of dendritic connections (Fig 3). It also correctly found a reduced number of osteocytes (osteocyte density) in the aged bone, as well as half the number of connections per osteocyte, in agreement with our manual measurements in our previous work. While an additional measure of average dendrite thickness was quantified, and no difference was found between young and aged mice, this measure may be much more sensitive to user error and image quality and so was significantly less reliable.

**Fig 4:**
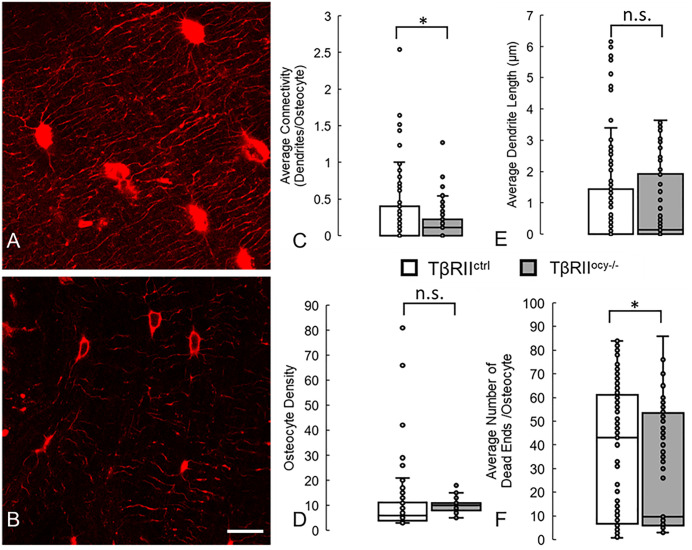
Deep learning model can detect and identify the lower osteocyte connectivity of bones in which TGF-β Receptor II has been ablated from osteocytes, compared with control mice. Some of these results (decreased connectivity and number of dead ends) are similar to those measured by us previously, while others (decreased osteocytes and dendrite length) are not fully captured [19]. (A) Confocal laser scanning microscopy images of osteocytes (red, phalloidin, cytoskeleton) from 2 month old control mice (TβRII^ctrl^) and (B) their litter-mates in which TGF-β Receptor II was ablated from osteocytes (TβRII^ocy-/-^). Running the trained deep learning model on these datasets correctly measured that aging causes significant decreases in average network connectivity and average number of blunted canaliculi in the network, but not previously observed decreases in osteocyte density and average dendrite length. * denotes *p* < 0.05 by Mann-Whitney U test compared to control, n.s. denotes no statistical significance. Scale bar = 10 µm and applies to all panels.

Similarly, our deep learning model could partially predict the degeneration in the osteocyte network induced by ablation of TGF-β Receptor II in osteocytes (Fig 4), capturing the trends of decreased connectivity and greater blunted canaliculi that we measured manual in our previous study [19]. However, the model did not correctly capture the decreases in osteocyte density and dendrite length that we found previously. This may indicate that training on larger datasets of osteocyte networks from healthy bone is required to pick up the more subtle degeneration driven by individual genetic mutations compared to the broad degeneration generated by aging.

### Deep learning models predicts similar changes to osteocyte network architecture to manual segmentation and measurement

**Fig 5:**
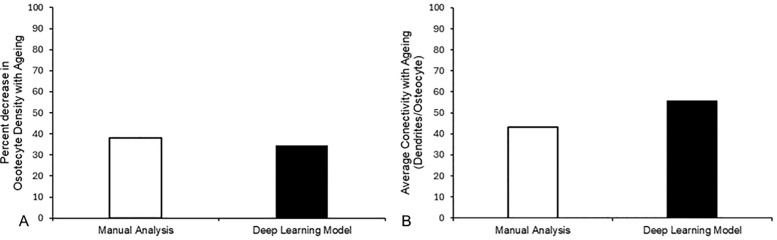
Deep learning model predicts changes in osteocyte network parameters due to age with similar accuracy to measurements performed manually. Direct comparison of measurements of percentage decrease in (A) osteocyte density and (B) osteocyte network connectivity as calculated manually by us previously [19], and by our deep learning model. Very similar decreases in osteocyte density were measured (38.03% vs. 34.44%, respectively) while a trend of similar results were found in connectivity of the osteocyte network (43.3% vs. 55.57%, respectively).

Direct comparisons between manual segmentation and quantification of the osteocyte network in 3D with the predictions of the model are challenging. Nonetheless, when investigating the percentage changes in parameters with ageing calculated by each method, the deep learning model calculates a similar 34.44% decrease in osteocyte density to that of 38.03% measured manually (Fig 5). Similarly, the decrease in osteocyte connectivity, or canaliculi per osteocyte, observed in the older mice was calculated to be 55.57% by the deep learning model, compared to a 43.3% drop measured manually.

Taken together, these results demonstrated the capacity of deep learning models, and the Attention U-Net in particular, to automatically characterise the age and integrity of the osteocyte network, providing a powerful new tool to analyse bone structure at the cellular scale.

## Discussion

This study presents, for the first time, a deep learning model capable of automatically segmenting and characterising the osteocyte network from microscopy scans. Testing a range of deep learning techniques, we identify Otsu as an effective thresholding method, and an Attention U-net as the most effective convolutional neural network that we tested for this task. In doing so, our model demonstrated capability to segment osteocytes and dendrites with 81.8% and 41.2% accuracy, respectively. Importantly, this model successfully distinguishes between the osteocyte networks of young and aged bone, matching our previously published results and validating the model [19].

In taking on such a challenging computational task, a number of limitations were necessary [33]. Primarily, the restricted size of our dataset (in terms of number of images and number of mice) appeared to be a pivotal constraint, leading to the model’s plateauing performance and resulting in fragmented dendrite segmentations and occasional misclassifications of osteocytes. The dataset comprised mice of significantly different ages (i.e., either end of the mouse lifespan) and this limited dataset also affected our quantitative assessments of osteocyte connectivity. Moreover, there is an absence of a benchmark dataset on the topic, making it challenging to compare our segmentation model or connectomics analysis methodology with prior endeavours outside our own previous works. To enhance our outcomes, expanding the dataset is an evident avenue of further enquiry. Indeed, other open-source datasets captured for different analysis methods are available and could be combined for additional comparisons [28]. Furthermore, while the manual annotation tool we utilised supported the resolution of our original images, the actual resolution proved somewhat inadequate for the precise annotation of thin structures. The dendrites typically had a pixel thickness ranging from 1.5 to 3 pixels, making their annotation exceptionally meticulous and challenging. Consequently, even minor discrepancies in annotations could lead to considerable variances in results. While utmost care was exerted to ensure the highest possible accuracy, including manual annotations by an expert operator with >15 years of experience of osteocyte segmentation, inter-operator variability may affect results and the practical challenges of zooming in and out, combined with the dendrites’ innate thinness, meant that some degree of imprecision was unavoidable. Furthermore, additional future research could study inter-observer error, which could be high for the dendrites. To mitigate the potential inconsistencies arising from this, we implemented an experimental strategy: a morphological dilation of the dendrite labels to slightly enhance their thickness (2x2, and 3x3 kernels). By doing so, we aimed to ensure a more accurate encapsulation of the dendrites as observed in the original images. Given the inherently slender nature of dendrites, we postulated that it would be beneficial to slightly overestimate their thickness in the labels rather than underestimate it. However, this is a limitation that should be addressed as models are further developed in future.

It is interesting that in using a relatively simple comparison of thresholding methods, we have shown that a well-known thresholding operation in biomedical image analysis, Otsu, is both a simpler and more accurate method than many of those applied previously. In particular, an accepted laborious task in the study of osteocytes is the manual segmentation of all of these individual cell bodies and dendrites for the study of their biochemistry, biomechanics, mechanobiology, etc. As previous methods had a significant manual component, the thresholding method we have studied could significantly simplify the preparation of osteocyte CLSM scans for many researchers. It should also be noted that we did not investigate the use of various image pre- and post-processing techniques (e.g., artifact detection, stain normalisation, patch selection), but evidence from numerous other biomedical and clinical applications suggest that these methods would provide yet greater increases in accuracy [60]. Indeed, previous work focused on LCN segmentation have shown that these techniques do improve the quality and performance of thresholding methods [31]. The further development of these techniques to automate the segmentation process, even at the current accuracy, would massively reduce what is currently an extremely labour-intensive task that relies on expert operators with years of training.

A particular challenge for these models appears to be accurate segmentation of dendrites, with our model achieving less than 50%. This may occur due to the convoluted trajectories of individual dendrites and canaliculi through the bone matrix in 3D, meaning that a confocal plane is highly unlikely to capture the path in its entirety. This limitation is inherent in both manual and automatic segmentation of the network, and confounds attempts to measure the true accuracy of algorithm compared to the actual network in vivo. Larger datasets of z-stacks, perhaps with narrower slice distances, will likely be required to overcome this technical challenge in future work. Additionally, although confocal microscopy is a popular tool for analysing the osteocyte network, it has intrinsic limitations at the wavelength limits of light, with some network structures being below this diffraction limit. However, our deep learning model confirmed that confocal microscopy has sufficient resolution to successfully capture changes on overall network topology, and as AI models usually improve significantly with additional data and only a few hundred 2D images were used in this study, this could likely be significantly improved with additional large datasets. Furthermore, as both more data and more training are clearly required, with larger datasets and matched resources for segmentation, this analysis could be extended to 3D across z-stacks in order to capture changes in the osteocyte network in its entirety. This would likely improve the accuracy of the deep learning model compared to manual operators, and would allow more direct comparisons to be made. Another notable result is the lack of change measured in dendrite or canalicular thickness, because if this parameter could be measured, then it would provide a significant boost to distinguish spatial differences in remodelling around osteocyte cell bodies or dendrites in the challenging new research area of periacunar-canalicular remodelling (PLR). This insensitivity to thickness changes was already a challenge due to the limitations of the wavelength of light, which is generally larger than the thickness of these changes. However, this is a challenge to which unsupervised machine learning may be well suited, as this type of pattern recognition is much better suited to computer vision and similar mapping of features has been performs on neurons in the brain [61]. This holds forth the tantalising prospect that future work could include unsupervised learning methods to enable us to actually measure the changes in canalicular and lacunar width associated with PLR.

Most excitingly, this first attempt to apply AI and computer vision to map the osteocyte network is already capable of distinguishing between the connectomics of young and aged bone to a very high degree of confidence. Additionally, it could measure a number of degenerative patterns induced by genetic modifications known to disrupt the osteocyte network, indicating that additional training could further improve this model as a tool for osteocyte analysis. Indeed, if trained on tightly controlled dataset of bones of across a range of ages, the model would well infer the age and condition of the osteocyte network in a given bone based solely on a confocal scan. A number of key morphological parameters measured by the model matched those previously quantified by our team using a manual image processing and connectomics approach [19], and thus this vastly improves the speed at which osteocyte network integrity can be quantified for these specific parameters. Therefore, in summary, our deep learning model represents a powerful new research tool, of immediate value to the researchers in multiple fields of bone research.

## Supporting information

S1 Fig Intra- and inter-operator variability comparing three repeat segmentations of a scan, and three separate scans, respectively. Expert operator was Operator A, while the trained operator was Operator B. Accuracy compared using the Dice Coefficient across the relevant scan.
(DOCX)

S1 Appendix Pseudocode of algorithm used to incorporate additional means of regularisation on the loss function. (DOCX)
