# Supplementary material for: Deep learning models to map osteocyte networks from confocal microscopy can successfully distinguish between young and aged bone
Source: PLoS Comput Biol. 2026 Jan 27;22(1):e1013914. doi: 10.1371/journal.pcbi.1013914 (PMC12875574; doi:10.1371/journal.pcbi.1013914)
Supplement: S1 Fig — Expert operator was Operator A, while the trained operator was Operator B. Accuracy compared using the Dice Coefficient across the relevant scan. (DOCX) [file pcbi.1013914.s001.docx]

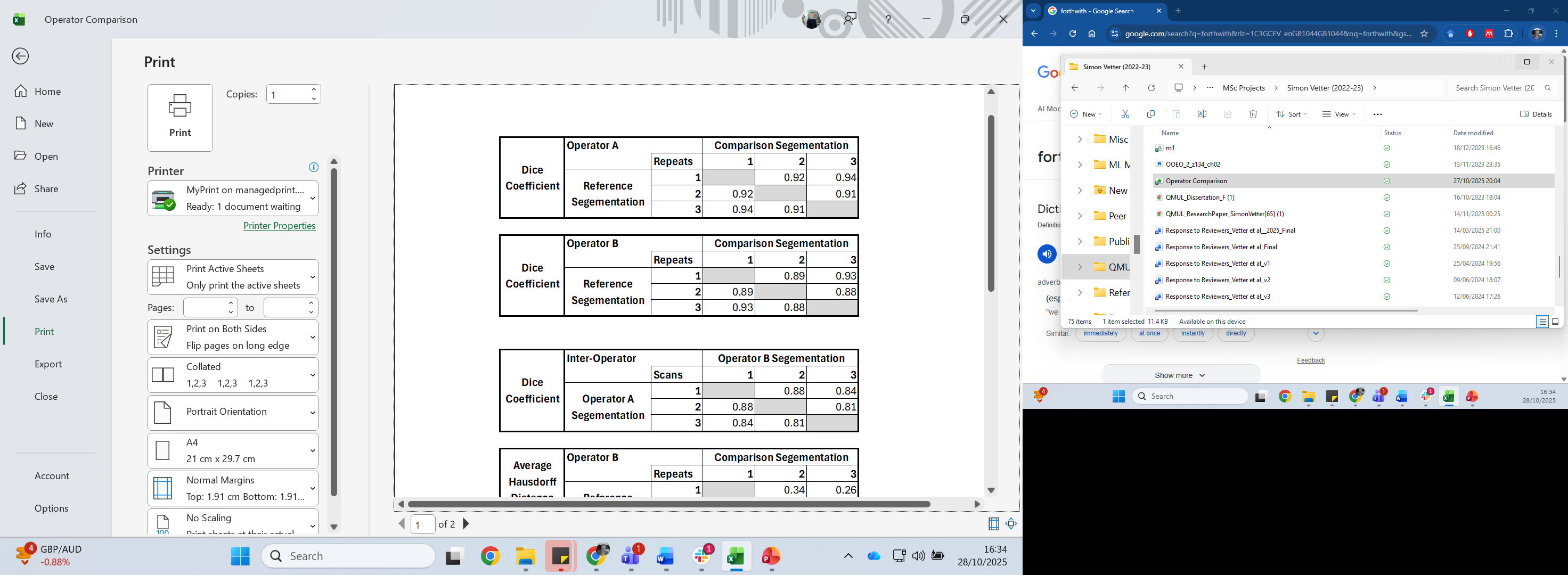


**S1 Fig: Intra- and inter-operator variability comparing three repeat segmentations of a scan, and three separate scans, respectively.** Expert operator was Operator A, while the trained operator was Operator B. Accuracy compared using the Dice Coefficient across the relevant scan.
