# Supplementary material for: Deep learning models to map osteocyte networks from confocal microscopy can successfully distinguish between young and aged bone
Source: PLoS Comput Biol. 2026 Jan 27;22(1):e1013914. doi: 10.1371/journal.pcbi.1013914 (PMC12875574; doi:10.1371/journal.pcbi.1013914)
Supplement: S1 Appendix — (DOCX) [file pcbi.1013914.s002.docx]

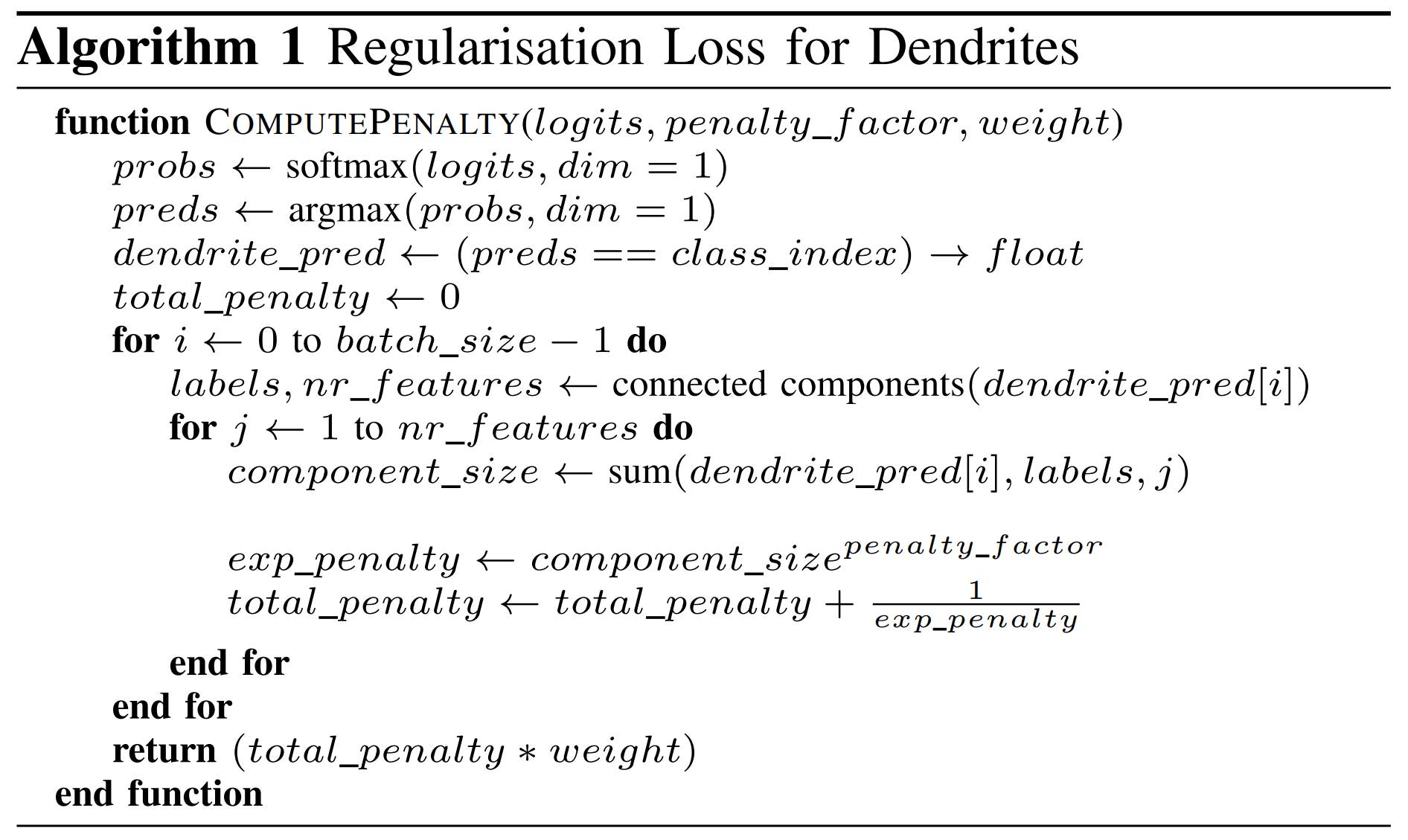


**S1 Appendix: Pseudocode of algorithm used to incorporate additional means of regularisation on the loss function**
